# Strength and Microstructure Characteristics of Blended Fly Ash and Ground Granulated Blast Furnace Slag Geopolymer Mortars with Na and K Silicate Solution

**DOI:** 10.3390/ma15010211

**Published:** 2021-12-28

**Authors:** Mateusz Sitarz, João Castro-Gomes, Izabela Hager

**Affiliations:** 1 Chair of Building Materials Engineering, Faculty of Civil Engineering, Cracow University of Technology, 31-155 Cracow, Poland; mateusz.sitarz@pk.edu.pl; 2 Centre of Materials and Building Technologies (C–MADE), Department of Civil Engineering and Architecture, University of Beira Interior (UBI), 6201-001 Covilhã, Portugal; jpcg@ubi.pt

**Keywords:** geopolymer, sodium and potassium silicate solution, microstructure, sustainability

## Abstract

Mineral geopolymer binders can be an attractive and more sustainable alternative to traditional Portland cement materials for special applications. In geopolymer technology the precursor is a source of silicon and aluminium oxides, the second component is an alkaline solution. In the synthesis of geopolymer binders the most commonly used alkaline solution is a mixture of sodium or potassium water glass with sodium or potassium hydroxide or silicate solution with a low molar ratio, which is more convenient and much safer in use. In this paper, we present the influence of sodium or potassium silicate solution on the physical and mechanical properties of fly ash and ground granulated blast furnace slag-based geopolymer mortars. Mercury intrusion porosimetry and microstructural observation allowed for comparing the structure of materials with a different type of alkaline solution. The evolution of compressive and flexural tensile strength with time determined for composites using 10%, 30% and 50% slag contents (referring to fly ash mass) was analysed. The tests were performed after 3, 7, 14 and 28 days. It was observed that, as the amount of slag used increases in the precursor, the strength of the material grows. Mortars with the sodium alkaline solution were characterised by a higher strength at a young age. However, the values of strength 28 days were higher for geopolymers with potassium alkaline solution reaching 75 MPa in compression. Geopolymer mortar microstructure observation indicates a high matrix heterogeneity with numerous microcracks. Matrix defects may be caused by the rapid kinetics of the material binding reaction or shrinkage associated with the drying of the material.

## 1. Introduction

Geopolymers are inorganic polymeric materials that have an amorphous or semi-crystalline structure and are similar to zeolites in chemical composition. For the production of geopolymer materials, a precursor is used, which is aluminosilicate material with high contents of silicon and aluminium oxides and an alkaline solution of Na (sodium) or K (potassium) silicates and hydroxides. Initially, geopolymers were developed as more temperature-resistant alternatives to thermoset polymers, but they are also currently being developed as construction materials with various applications [1].

A cement-free binder is one of their applications, in which Portland cement (PC) is replaced to manufacture a high-quality matrix for construction materials: geopolymer concretes and mortars. The use of geopolymer binders reduces CO_2_ emissions associated with Portland cement production. In addition, aluminosilicate precursors, which are industrial waste [2] such as FA (fly ash) and GGBFS (ground granulated blast furnace slag) or mining tailings or waste andesite dust [3] are often used in the process of geopolymer synthesis.

FA is a by-product produced in coal-fired power plants. The composition of this waste material is significantly variable, depending on the coal source and burning conditions. The decisive factors that control the reactivity of the precursor and the solubility in alkaline solutions are the chemical composition, the contents of the crystalline and glass phases and the particle size distribution. The results of the research indicate that the microstructure of the geopolymer is particularly influenced by the particle fineness, the composition of the amorphous phase and the content of oxides [4]. Blast furnace slag is formed as a by-product of iron ore smelting. It is calcium-rich raw material, used in the production of mineral binders. Ground granulated blast furnace slag is a mixture highly glassy phases with a composition close to that of gehlenite and akermanite [4].

The properties and durability of composites based on geopolymer binders are sometimes even better than those obtained with Portland cement [5]. High compressive strength is associated with the interconnected structure of oligomers [6,7].

Such materials present beneficial properties in harsh environmental conditions such as acid and sulphate attack, chloride ingress and thermal and fire conditions (T = 1000–1200 °C) [7,8]. Moreover, geopolymer matrix materials may undergo rapid setting, still presenting suitable long-term strength [6].

The geopolymers show a great potential also in 3D printing manufacturing both in additive manufacturing, where specific rheological performances need to be ensured [9,10], but also in a powder-based 3D printer dry geopolymer-based material achieved satisfactory results [11].

The mechanisms of geopolymer products formation are complex. According to [12], the geopolymer synthesis process consists of the following phases: alkalinization, depolymerisation of silicates, gel formation of oligo-sialates, polycondensation, reticulation, networking and geopolymer solidification. If these reactions are to proceed correctly, they require the supply of aluminosilicate raw materials with a high content of SiO_2_ and Al_2_O_3_ and the appropriate amount of alkali metal Na or K ions in an alkaline solution [13]. An alkaline medium is required to enable silica and alumina dissolving and also to ensure hydrolyse of the raw material particle surfaces [12,14]. Scientific literature related to geopolymers and alkali-activated materials (AAM) offers a number of possibilities regarding the type of alkaline solution to be used and the proportion of alkali species such as hydroxides and/or silicates in the activating solution. The typically used solution is a mix of liquid silicates and hydroxides (solids dissolved in water) [13]. The commonly used liquid silicate solutions have a molar modulus of about 2.5. The molar modulus (molar ratio) or silicate modulus, is the basic characteristic of aqueous solutions of sodium or potassium silicate equal to the ratio of the number of moles of silicon oxide to moles of metal oxide. The synthesis process of geopolymeric materials is much more effective when using solutions with a modulus below 2.0. Hence, it is necessary to combine silicate solutions with hydroxide. The purpose of this combination is to lower the value of the molar modulus of the alkaline mixture. In the conducted research, we used Geosil^®^ ready-to-use products with a molar module of 1.7, dedicated to the geopolymer binder. This solution is convenient in the application and safer for the user. It reduces the troublesome process of mixing alkaline substances and improves the process of preparing a geopolymer mix. Such technological facilitations also extend the possibilities of using geopolymer materials [15]. As alkali-activators have been suggested to be a contributor to the environmental burdens due to the energy-demanding production process, there are studies showing the possibilities to produce alkali-activated materials from chemically modified waste-derived activators: waste glass and rice husk ash [16] therefore, the environmental impact of geopolymers can be significantly reduced.

Another important factor in the technology of geopolymer materials is curing parameters, such as the curing temperature and moisture exchange with the surroundings. For FA-based geopolymers, thermal curing is necessary for setting and geopolymerisation to occur [17]. This is considered an important limitation for the application of those materials. Furthermore, the need to heat the fly ash geopolymers increases the energy consumption of the entire process. Using Ca-rich raw material such as ground granulated blast-furnace slag in combination with fly ash, as the former reacts at room temperature, gives promising results in the production of calcium–aluminium–silicate–hydrate (C–A–S–H) gel.

Moreover, the reaction develops quickly, which means a rapid initial setting time. Previous results have shown that the blend of FA–GGBFS [18,19] reacts at room temperature. Changing the ratio between FA and GGBFS allows adjusting the setting time. This requires further research to provide more data to better understand the geopolymer binding mechanisms of such binary systems.

This research presents an investigation into how the low sodium or potassium silicate molar ratio solution affects properties of the blend of FA- and GGBFS-based geopolymer mortars. During geopolymerisation, the alkaline solution plays a crucial role in the material mechanical properties development. The most important characteristics of an alkaline solution are (1) proportions between the hydroxide and the silicate, (2) hydroxide concentration [20], (3) type of cations [21] and (4) the molar ratio of the alkaline solution. All these parameters affect the mechanical behaviour of geopolymers. The main goal and ambition of this study is to show the impact of the silicate solution type (sodium or potassium) on the microstructure development, porosity and mechanical properties of mortars with geopolymer matrix. A one-component alkaline solution was used in this study, which facilitated the geopolymer mixture preparation process, limiting the arduous hydroxide dissolution procedure.

## 2. Materials and Methods

### 2.1. Raw Materials

The precursor for this research was composed of fly ash, which is waste from coal combustion, containing an abundance of silica and alumina (SiO_2_ and Al_2_O_3_). The oxide composition of FA is shown in Table 1; the FA specific mass was 2.1 g/cm^3^. Following the ASTM C618 document [22], this fly ash can be classified as F class ash. The FA also meets the standard EN 450-1:2012 [23] requirements for concrete additive type II.

The second component of the precursor was ground granulated blast furnace slag. This CaO-rich raw material makes it possible for the mixture to bind at ambient temperature. The chemical composition was specified by the supplier, and the detected oxides are presented in Table 2. The GGBFS specific mass was 2.9 g/cm^3^.

Commercially available liquid silicates were used as the alkali source. Sodium silicate (Na-Sil) and potassium silicate (K-Sil) were used. The molar ratio (MR) of liquid silicates is the activating solution mole ratio of silica to sodium oxide or to potassium oxide. The MR of 1.7 was the same for both solutions. Their chemical specifications are presented in Table 3.

In this study, the ratio between the total water mass (i.e., sum of mass of water added and mass of water in the liquid silicates) and the total mass of binder is the water/binder ratio. The water/binder ratio was kept constant at 0.30 for all mortars. In these considerations the binder mass is the sum of FA and GGBFS masses. The amount of additional water necessary to obtain the workable mixture was determined based on previous research [24] and the extra water was added directly to the liquid silicates. Quartz sand was used (d = 0/2 mm; specific mass of 2.64 g/cm^3^) for preparing all the geopolymer mortars. The particle size distribution of quartz sand is given in Table 4 corresponded to standardized curve for sands used for mortars (CEN Standard Sand according to EN 196-1 [25]. 

The sand to binder mass ratio was kept constant, with a value of 1.50. For all materials, the mass ratio of the FA, GGBFS and sand was the same. Three levels of GGBFS addition were used: 10%, 30% and 50% of FA mass. 

To assess the influence of the sodium or potassium silicate solution on the properties of the geopolymer mortars, three mortars were manufactured. Two types of liquid silicates were used to prepare each mortar: K-Sil and Na-Sil, which are potassium silicate solution and sodium silicate solution, respectively. The details on the mix compositions are in Table 5.

The differences in density between FA and GGBFS required mass shares of other components to be adjusted. This enabled to obtain the same volume of mortar for different ratios of fly ash and ground granulated blast furnace slag masses.

### 2.2. Preparation of Mortars and Samples

Geopolymer mortars were produced according to the procedure which first involves the preparation of the binder. First, supplementary water was added to the liquid silicates, which is required to obtain the water/binder ratio. Subsequently, FA was mixed with the alkaline-activating solution. Mixing was performed in a rotary mixer for 10 min at a low speed. In the next step, ground granulated blast furnace slag was added and 5 min mixing was applied. In this way, the geopolymer binder was obtained. While mixing the binder, the rotary mixer was switched off for 1 min to remove the solids stuck to the walls of the container. Finally, sand was gradually added to the mixture for 3 min, while the rotary mixer worked at low speeds. The total mixing duration time was aprox. of 20 min it has been selected based on literature reports [12,26] and optimised as the result of the team’s previous experience in the preparation of geopolymer blends. This mixing time guarantees the homogenization of the mixture of precursor and activator. Shorter mixing periods should be applied when a high amount of GGBFS is used in FA blended geopolymer due to the flash-setting in mixes where a high CaO amount is provided [27,28].

The geopolymer mortars were moulded into prismatic 40 × 40 × 160 mm specimens. The samples were compacted on a shaking table and covered with plastic lids. Twenty-four hours after casting, the specimens were removed from moulds and stored at room temperature (18 °C ± 2 °C) and relative humidity HR = 75%, protected from water evaporation using plastic to limit moisture exchange with the environment.

### 2.3. Mechanical Tests

Bending and compressive strength tests were performed in a mechanical device (CONTROLS, construction materials testing equipment). For bending, two 40 × 40 × 160 mm prismatic specimens at each age (3, 7, 14 and 28 days) were tested for each mortar type. The loading rate applied was of 50 N/s, as this is recommended in PN-EN 196-1 for testing cement mortars. Apart from the bending test, compressive strength analysis was also carried out on the samples of the same age. The mortar prisms used in these tests were the ones remaining from the bending test. During the compressive tests, the loading rate was 2400 N/s, as this is also the adequate value according to PN-EN 196-1.

### 2.4. SEM and MIP

Scanning electron microscopy (SEM, Zeiss EVO-MA 10) observations and mercury intrusion porosimetry (MIP, PoreMaster 33 Automatic Pore Size Analyzer made by Quantachrome) were used for microstructure analysis and to evaluate total porosity, pore size distribution and volume of pores. SEM observations, as well as an EDS chemical analysis, and MIP were carried out for mortars with 50% FA and 50% GGBFS blended binders. After 28 days of curing, porosity (MIP) and SEM observations were conducted.

## 3. Results and Discussion

### 3.1. Mechanical Parameters

**Figure 1:**
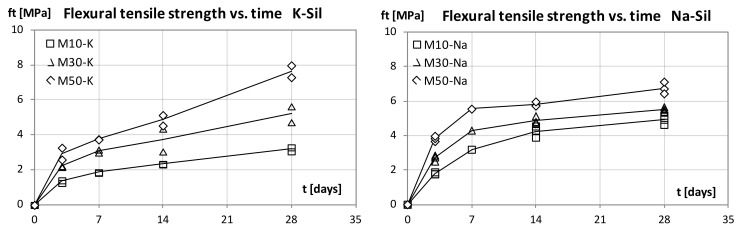
Flexural tensile strength developmnt with curing time, K-Sil and Na-Sil.

**Figure 2:**
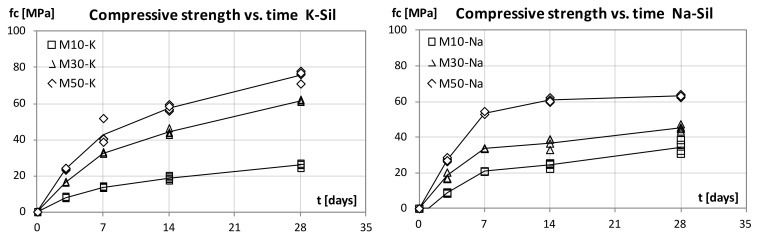
Compressive strength development with curing time, K-Sil and Na-Sil.

Our examination focused on the development of the mortar strength parameters and its bending and compressive strength. The Figure 1 and Figure 2 show the results. Regardless of the alkaline solution type used, the strength increased along with the amount of slag in the binder formula. Twenty-eight days after casting, the highest flexural and compressive strength values were observed in materials with a 50% GGBFS content and the potassium alkaline solution. The recorded values were 7.7 MPa for bending and 75.4 MPa for compression. Compared to the mortar made with the sodium alkaline solution, the values were 20% higher for compressive strength and 15% higher for bending strength. After 28 days, the values of flexural strength ranged from 10% to 14% of the 28-day compressive strength for mortars with the sodium alkaline solution and from 8% to 10% for materials with the potassium solution. The higher strength of the potassium-based materials indicates a beneficial effect of K+ ion on the formation of geopolymerization products. The presence of potassium cations promotes the formation of aluminum silicate gel on the surface of the fly ash particles. Compared to sodium ions, potassium cations create a denser structure of the material and the bonds between the mineral precursor molecules and the alkaline activator are stronger. Such effect of a potassium alkaline solution is reported in the literature. The size of metal cation K+ (1.33 Å) is larger than that of Na+ ion (0.97 Å) which results in formation of compact structure in the geopolymer matrix. On the chemical side, the cation K + should be associated with more water molecules than the cation Na + which results in an increase in the condensation step in the geopolymerization reaction during the formation of amorphous alumina-silicate structures [29,30,31].

Materials with different types of alkaline solutions also demonstrated different kinetics of the strength increase. Mechanical test results after 3 and 7 days show higher strength of sodium-based materials. The trend changes later. After 14 days, the strength values (especially the compressive strength) were similar regardless of the type of alkaline solution used. Regardless of the GGBS dosage, between days 14 and 28, a much higher dynamic of strength increase was observed in mortars with the potassium alkaline solution. 

Differences in the development of strength may be related to the different size of the sodium and potassium cations. Smaller sodium ions can move more easily in the geopolymer matrix so that the sodium activator can release more silicate and aluminate monomers [32]. It is the reason why, in the first phase, the sodium materials can obtain higher strength. After an extended period of time, the effect of the particle size begins to change. Larger potassium cations allow to form a stronger mineral matrix [29].

It is worth noting that 7-day values of compressive strength are high. They amount to 85% of the 28-day value for mortars with the sodium alkaline solution and 50% GGBFS content. This result is higher compared to cement-based materials. Similar trends confirming the rapid increase in the strength of geopolymer materials were observed by Mehta et al. [33]. The geopolymer binders’ high strength at an early age opens the possibility of using these materials in structural repairs, where the dynamics of strength increase are important.

### 3.2. SEM Analysis

**Figure 3:**
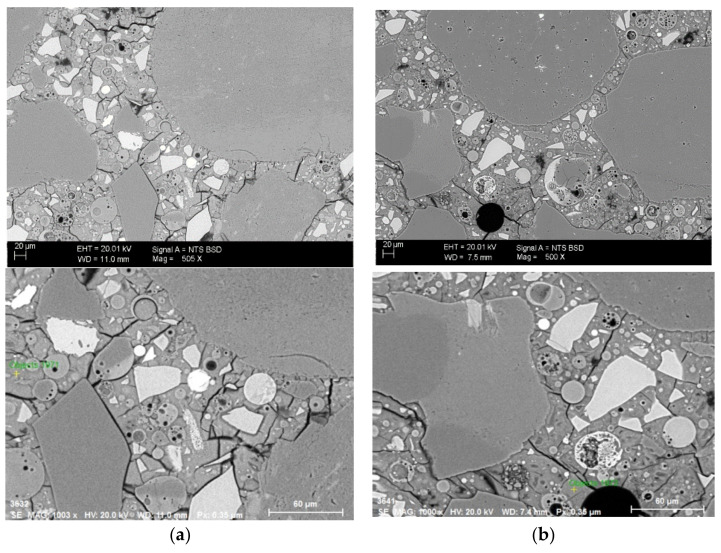
SEM images of geopolymer mortars (**a**) M50-K and (**b**) M50-Na.

Our own observations are presented below. Figure 3 shows the micrographs of the samples with potassium silicate (M50-K) and sodium silicate (M50-Na).

Both samples have a similar microstructure in which we can observe unreacted particles of FA and grains of GGBFS surrounded by a matrix that is not homogeneous in many places. There are relatively numerous fractures in the matrix. Both mortars with a high GGBFS content had similar short setting times. The heterogeneous matrix can be associated with the fast geopolymer reaction kinetics or shrinkage caused by drying of the material. In the interfacial transition zone (between the aggregates and geopolymer matrix) of the mortars, the fine aggregates are surrounded by cracks in some places.

Table 6 shows EDS-based chemical analysis of the geopolymer matrix with 50% FA or 50% GGBFS with potassium or sodium silicate. The composition of the matrix indicates the presence of the phase characteristics of blended FA–GGBFS geopolymer binders. Sodium (N–A–S–H) and potassium (K–A–S–H) alumina silicate hydrate gels and calcium alumina silicate hydrate gel (C–A–S–H) improve all the mechanical performances of the material. The cohabitation of both gels N–A–S–H and C–A–S–H has been discussed widely in other publications [34,35].

### 3.3. Mercury Intrusion Porosimetry

**Figure 4:**
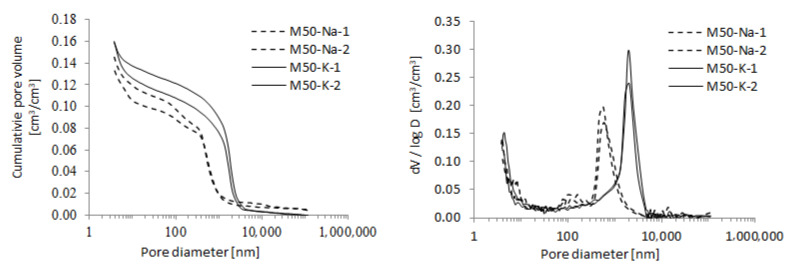
The cumulative pore volume and pore size distribution for binder containing 50% FA and 50% of GGBFS.

The distribution of pore size was measured with mercury intrusion (MIP). This method allowed for the detection of open pores with the size of 3.5 nm to 500 μm. Two specimens of mortars were prepared with binders containing 50% FA and 50% GGBFS. The results are depicted in Figure 4. The total porosity was read from the curves in the cumulative intrusion volume.

**Figure 5:**
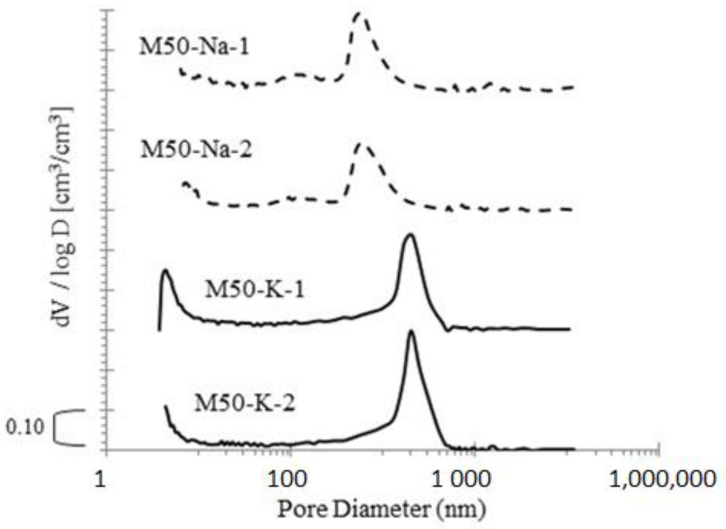
Comparison of critical pore diameter for M50-K and M50-Na materials.

The MIP parameters obtained for two specimens of M50-K and M50-Na are summarized in Table 7. The total porosity, average pore diameter and critical pore diameter was read from the curves in the cumulative intrusion volume. The Figure 5 compares the critical pore diameter obtained for M50-K and M50-Na materials. 

The cumulative intrusion of mercury curves for both types of materials are similar. The total porosity mean values were of 0.142 and 0.159 cm^3^/cm^3^ for M50-Na and M50-K, respectively. The use of the potassium silicate solution resulted in a slightly higher porosity. In other publications on the subject, we can find results showing that the pore size distribution of geopolymers resembles a typical bell curve centred in the region where mesopores occur. 

Curves for both tested mortars showed one clear peak bell shape. The peaks were situated near 600 nm and 2000 nm for M50-Na and M50-K, respectively. Additionally, mortars with the sodium alkaline solution had more pores in the range of 100 nm. For materials with the potassium alkaline solution, the peak was higher and moved towards larger pore diameters.

**Figure 6:**
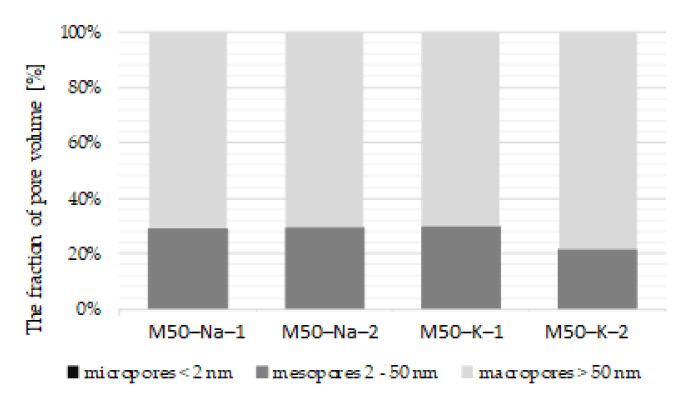
Types of pores (micro-, meso-, macro-) for M50-K and M50-Na materials.

The Figure 6 shows the porosity structure of the materials with specific types of pores according to the criteria proposed by IUPAC (International Union of Pure and Applied Chemistry). Due to the limitations of the Mercury Porosimetry method, it was not possible to determine the share of micropores (<2 nm) in the total porosity of the material. The part of both meso and macro pores in all tested materials is at a similar level. The porosity structure of M-50-Na and M-50-K materials is dominated by macropores (>50 nm) which constitute 70% of the total porosity. The mesopores (2–50 nm) share is about 30%.

## 4. Conclusions

This study focused on the examination of the mechanical properties of FA–GGBFS blended geopolymer mortars. Mortars that contained three different levels of GGBFS, activated with the sodium or potassium alkaline solution, were compared. All prepared materials were set and hardened at ambient temperature with no additional heating. For the geopolymer mortars with blended binders, the compressive strength at 28 days was higher than 75 MPa for 50% FA and 50% GGBFS blended precursors with the potassium alkaline solution. We observed that, as the amount of GGBFS used increases, the strength of the material grows. Mortars with the sodium alkaline solution were characterised by a higher strength at a young age (3 and 7 days). However, the values of strength 28 days after casting were higher for geopolymer mortars with the potassium alkaline solution.

Observations of the blended FA–GGBFS geopolymer mortar microstructure indicate a high matrix heterogeneity with numerous microcracks. Matrix defects may be caused by the rapid kinetics of the material binding reaction or shrinkage associated with the drying of the material. In the analysed case, with a high content of GGBFS in the binder, there were no evidently visible differences between the alkaline solution types that we used. The structure of the material was primarily determined by the precursor that was used. The presence of phases N(K)–A–S–H and C–A–S–H in the material can be deduced from the matrix chemical analysis and EDS observations. The higher values of strength for highly dosed GGBFS were associated with the more compact and dense structure of C–A–S–H in the material. 

The results of the presented research provide information on the influence of the alkaline factor on the selected properties of the geopolymeric mortar with mixed precursor (FA-GGBFS). The observations resulting from the research allow to state that the type of the alkali metal cation significantly influences the structure of the created geopolymer matrix. Smaller sodium ions can move more easily in the geopolymeric matrix and more effectively initiate the geopolymerization reaction by more intensive release of silicate and aluminate monomers. On the other hand, larger potassium ions may be responsible for the formation of a strong geopolymeric backbone in the structure of the material. The binding mechanism of aluminosilicate materials in an alkaline environment is complex and still not fully understood. The authors of the research plan to extend the work carried out and a detailed structural analysis of the developed binders. 

The variety of raw materials used, as well as chemical and morphological differentiation cause difficulties in creating universal rules in geopolymer technology. For alkali activated materials it is most advantageous to use locally available waste materials. Therefore, it is very often necessary to develop technology taking into account the nature of the specific, available raw material.

## Figures and Tables

**Table 1:** XRF FA analysis.

SiO_2_	Al_2_O_3_	Fe_2_O_3_	CaO	MgO	SO_3_	K_2_O	Na_2_O	P_2_O_5_	TiO_2_	Mn_3_O_4_
52.30	28.05	6.32	3.05	1.71	0.28	2.51	0.76	0.69	1.35	0.07

**Table 2:** Ground blast furnace slag composition.

SiO_2_	Al_2_O_3_	Fe_2_O_3_	CaO	MgO	SO_3_	K_2_O	Na_2_O	Cl¯	Na_2_Oeq	Blaine (cm^2^/g)
39.31	7.61	1.49	43.90	4.15	0.51	0.356	0.468	0.038	0.702	3904

**Table 3:** Composition of Woellner Geosil liquid silicates, manufacturer data.

Content/Property	Unit	Geosil 34417 (Na-Sil)	Geosil 14517 (K-Sil)
Na_2_O	%	16.74	-
K_2_O	%	-	21.84
SiO_2_	%	27.5	23.5
Density	g/cm^3^	1.552	1.512
Viscosity	m Pa.s	470.0	22.0

**Table 4:** Particle size distribution of quartz sand.

Mesh Size (mm)	Average (%)
2.00	0
1.60	7
1.00	33
0.50	67
0.16	87
0.08	99

**Table 5:** Mix compositions of geopolymer mortars with sodium and potassium silicate solution.

Components	M10-Na	M30-Na	M50-Na	M10-K	M30-K	M50-K
	(kg/m³)					
Alkaline solution Na-Sil + water	333.9	340.6	347.5			
Alkaline solution K-Sil + water				331.7	338.3	345.1
FA	667.9	529.8	386.1	663.4	526.2	383.4
GGBFS	74.2	227.1	386.1	73.7	225.5	383.4
Sand (0/2 mm)	1113.2	1135.3	1158.3	1105.7	1127.5	1150.3

**Table 6:** EDS analysis of geopolymer matrix of M50-K and M50-Na.

Element	O	Si	Ca	C	Al	K	Na	Mg	Fe	
M50-K (matrix)	45.12	16.86	10.59	8.65	4.89	5.02	1.25	6.09	1.03	wt %
M50-Na (matrix)	44.81	20.51	14.10	10.37	4.49	0.77	2.76	1.37	0.81	wt %

**Table 7:** Mercury intrusion porosimetry (MIP) parameters for M50-K and M50-Na materials.

	M50-Na-1	M50-Na-2	M50-K-1	M50-K-2
Porosity (%)	14.9	13.4	15.8	16.0
Critical pore diameter (nm)	580.7	589.3	2095.0	1999.0
